# RNA-based therapies for neurodevelopmental disorders: innovative tools for molecular correction

**DOI:** 10.3389/fmolb.2025.1681647

**Published:** 2025-10-31

**Authors:** Denise Drongitis, Lucia Verrillo, Alberto de Bellis, Maria Giuseppina Miano

**Affiliations:** ^1^ Institute of Genetics and Biophysics “Adriano Buzzati-Traverso”, CNR, Naples, Italy; ^2^ Maria Rosaria Maglione Foundation Onlus, Naples, Italy; ^3^ Azienda Ospedaliera di Rilievo Nazionale e di Alta Specializzazione (A.O.R.N.) Sant’ Anna and San Sebastiano Hospital, Division of Neurosurgery, Caserta, Italy

**Keywords:** genetic NDDs, animal models, RNA metabolism, gene expression regulation, haploinsufficiency, translation

## Abstract

Modulation of RNA and protein expression to restore or normalize neuronal function has emerged as a powerful therapeutic strategy for neurodevelopmental disorders (NDDs) tailoring individual genetic mutations causing intellectual disability (ID), or autism spectrum disorder (ASD), or developmental epileptic encephalopathy (DEE). In recent years, diverse classes of RNA-based molecules have been developed with therapeutic potential, including antisense oligonucleotides (ASOs), oligonucleotides targeting natural antisense transcripts (antagoNATs), Short Interspersed Nuclear Element UP-regulating RNAs (SINEUPs), interfering RNAs (RNAi), Exon-Specific engineering U1 small nuclear RNAs (ExSpeU1s), and small-activating RNA (saRNA) This review highlights the promising advances of these RNA-based therapeutics in addressing syndromic ID, such as Fragile X syndrome, *MECP2* duplication syndrome, *FOXG1*-gene related Rett syndrome and Angelman syndrome, which are characterized by well-defined genetic mutations with limited treatment options. Moreover, ASD-related condition linked to mutations in *CHD8* is under investigation, extending the therapeutic landscape to complex behavioral and cognitive disorders. In the same way, several DEEs caused by mutations in *CDKL5*, *DNM1*, *KCNT1, SCN1A*, *SCN2A*, *SCN8A*, and *UBA5* genes, which present severe pharmaco-resistant epilepsy, are increasingly becoming targets for RNA molecules that aim to restore neuronal excitability and network function. Together, these findings underscore the expanding therapeutic landscape enabled by RNA technologies, offering unprecedented specificity and flexibility for gene-targeted interventions in NDDs. As the field of RNA medicine continues to evolve across genomics and neuroscience, we aim to provide a resource for researchers and clinicians on promising innovative tools for molecular correction.

## Introduction

Neurodevelopmental disorders (NDDs) comprise a diverse group of chronic conditions characterized by impairments in brain development and functioning (Parenti et al., 2020). They include intellectual disability (ID), autism spectrum disorder (ASD) and developmental and epileptic encephalopathies (DEEs). Typically manifesting during the early developmental stages, these disorders persist into childhood and adulthood, often associated with cognitive, behavioral, and neurological deficits. Despite advances in genetic and molecular underpinnings of NDDs, effective treatments remain limited. Many of the hallmark symptoms—such as cognitive delay, communication impairment and epilepsy are refractory to conventional pharmacological approaches. As a result, most NDDs are currently incurable, with available therapies focused primarily on symptom management rather than disease modification. This therapeutic gap underscores the urgent need for innovative, targeted interventions that address the underlying molecular dysfunctions. Therefore, a major goal of current research is to develop gene mutation-based strategies that can mitigate severe neurological symptoms with an important impact on disease cure and patient prognoses.

The widespread implementation of next-generation sequencing has significantly improved the identification of pathogenic NDD variants (Sullivan et al., 2023), paving the way for molecular targeted therapy. However, many disease-associated pathways remain undruggable or challenging to target using small-molecule approaches. These limitations have fueled growing interest in RNA therapeutics, a promising class of molecules that can directly restore deficient protein expression, or silence aberrant gene products in those pathogenic conditions driven by disease-causing transcripts (Anthony, 2022). This approach holds promise for monogenic NDDs arising from haploinsufficiency, gain-of-function (GoF) mutations, or splicing abnormalities, all of which can be effectively targeted through transcript-level modulation. Moreover, the inherent flexibility of RNA-based strategies—characterized by adjustable dosing and low genotoxicity—makes them especially well-suited for the development of innovative therapies for untreatable pediatric diseases.

This review delves into an emerging and highly dynamic field of research centered on RNA therapeutics, as precision tools for molecular correction in specific genetic conditions affecting brain development and functioning, such as ID, ASD, and DEE. These genetically heterogeneous conditions often involve dysregulated or aberrant gene expression that disrupt specific neurodevelopmental processes. In this context, RNA-based therapeutics have gained considerable attention recently due to their ability to modulate gene or protein expression without altering the underlying DNA sequence.

We here report a range of RNA modalities applied to a number of NDD conditions, including antisense oligonucleotides (ASOs), which reduce toxic mRNA transcripts; oligonucleotides targeting natural antisense transcripts (antagoNATs), which inhibit natural antisense transcripts and enhance expression of silenced genes; Short Interspersed Nuclear Element UP-regulating RNAs (SINEUPs), which upregulate translation of specific mRNAs through an antisense domain and a functional SINEB2 element; RNA interference (RNAi), which selectively degrade mutant transcripts; exon-specific engineering U1 small nuclear RNAs (ExSpeU1s), which rescue defective mRNA splicing patterns, and small-activating RNAs (saRNAs) that target gene promoters to induce transcriptional gene activation. Promising findings from preclinical studies and early-stage clinical trials highlight the significant potential of RNA-based approaches to influence the disease manifestations in various genetic forms of NDDs. Importantly, a few active clinical trials are on track marking a crucial advancement in the pursuit of tailored, gene mutation-based strategies for pediatric neurogenetic diseases.

## RNA modalities explored for NDD therapy

**FIGURE 1:**
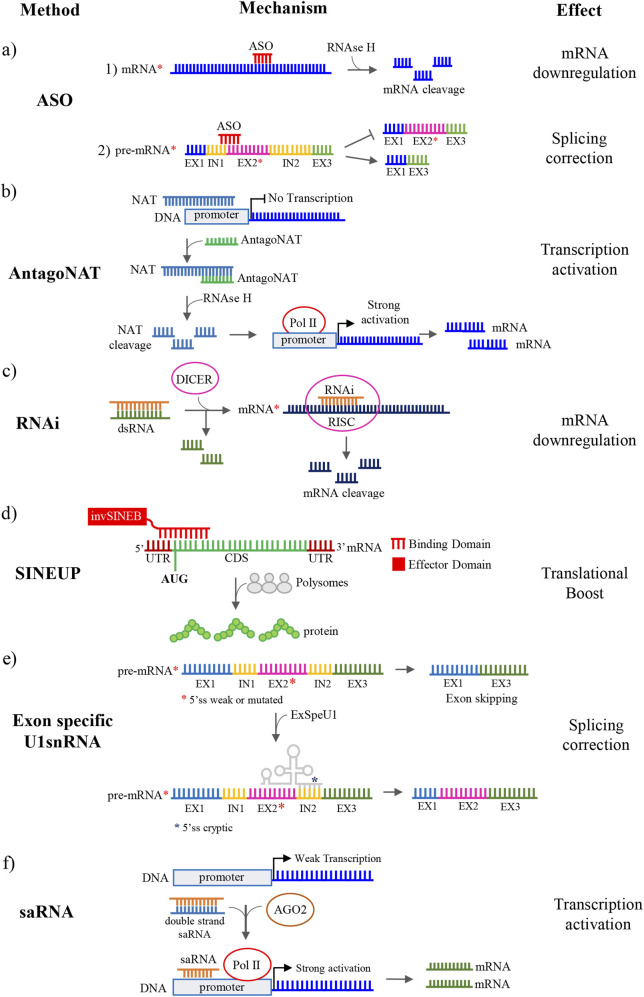
Strategies for RNA-based therapy in NDDs discussed in this review. **(a)** Antisense oligonucleotides (ASOs) modulate the target gene expression by triggering the mRNA cleavage by RNAse H or by modulating RNA splicing to induce exon skipping or exon inclusion; **(b)** Antagonist of natural antisense transcripts (antagoNATs) bind non-coding antisense RNAs inducing their degradation through the RNAse H activity. NATs degradation allows the binding of the POLII to the promoter sequence leading to a strong activation of mRNA transcription with resultant up-regulation at the protein level; **(c)** Small RNA interference (RNAi) is mediated by double-stranded RNA (dsRNA) molecules which are processed in small fragment (siRNA) by DICER. The antisense strand of siRNA (in *orange*) is loaded into the RISC complex for mRNA targeting and cleavage; **(d)** Short Interspersed Nuclear Element UP-regulating RNAs (SINEUPs) are antisense long non-coding RNAs (lncRNAs) with a dual-domain structure with a target-specific antisense region that binds to the 5′ untranslated region (UTR) of the mRNA, and an effector domain derived from an inverted SINEB2 element, which facilitates the recruitment of ribosomes boosting protein synthesis; **(e)** Exon-Specific engineered U1 small nuclear RNAs (ExSpeU1s) modulate the target gene expression by splicing correction. ExSpeU1s are designed to be complementary to 5’ss cryptic (*blue* asterisk) located in an intronic region downstream the mutant or weak 5’ss (*red* asterisk). The binding of ExSpeU1 to the cryptic 5’ss promotes the use of the mutated or weak 5’ss leading to exon inclusion; **(f)** small activating RNAs (saRNAs) are small molecules that bind the promoter of their target genes and thus regulate gene expression at transcriptional level. In details, a promoter-targeting duplex RNA is loaded onto AGO2 which cleaves the passenger strand and uses the remaining RNA strand as the guide to form an active RNA-Ago2 complex. After translocating into the nucleus, the RNA guide strand directs AGO2 to its promoter target, acting as a recruitment platform where different components are assembled to stimulate transcription initiation. *Red* asterisks indicate pre-mRNA or mRNA carrying mutations.

Different classes of RNA-based molecules have been designed as targeted therapies for precise modulation of transcript or protein expression in Fragile X Syndrome (FXS), *MECP2* duplication syndrome (MDS), Angelman syndrome (AS), *CHD8*-related ASD, and distinct genetic forms of DEEs (Table 1). Such precision is particularly valuable in these pathologies, where mutations with early-onset consequences can cause profound neural circuit dysfunction during critical periods of brain development. Timely intervention with allele-specific RNA strategy has the potential to alter disease manifestations by preserving network architecture and preventing cumulative neurological damage. Specifically, some approaches are designed to suppress the production of toxic or pathogenic proteins by promoting mRNA degradation or inhibiting translation. This is typically achieved using ASOs or RNAi technologies (Figure 1). In contrast, other strategies aim to boost the expression of functional proteins by stabilizing mRNA as antagoNATs, or enhancing its transcription or translation, such as saRNA and SINEUPs, respectively (Figure 1). Furthermore, correcting defective mRNA splicing to restore proper protein structure is accomplished through the use of splice-switching ASOs and ExSpeU1s (Figure 1).

**TABLE 1:** Summary of RNA-Based therapeutic approaches for NDDs discussed in this review.

RNA modality	Gene target	Disease/condition	ClinicalTrials Gov ID	References
ASO	*FMR1*	Fragile X syndrome (FXS; MIM 300624)	nd	Shah et al. (2023)
	*MECP2*	*MECP2* duplication syndrome (MDS; MIM 300260)	nd	Shao et al. (2021); Bajikar et al. (2024)
	*SCN1A*	Dravet syndrome (DS; MIM 607208)	NCT04740476	Yuan et al. (2024)
	*SCN2A*	Developmental and epileptic encephalopathy 11 (DEE11; MIM 613721)	NCT07019922	Li et al. (2021); Wagner et al. (2025)
	*SCN8A*	Developmental and epileptic encephalopathy 13 (DEE13; MIM 614558)	nd	Hill et al. (2024)
	*KCNT1*	Developmental and epileptic encephalopathy 14 (DEE14; MIM 614959)	nd	Burbano et al. (2022)
AntagoNAT	*UBE3A*	Angelman syndrome (AS; MIM 105830)	NCT04259281 NCT04428281 NCT05127226	Clarke et al. (2024)
SINEUP	*CHD8*	Autism spectrum disorders (ASD; MIM 615032)	nd	Di Leva et al. (2025)
	*UBA5*	Developmental and epileptic encephalopathy 31 (DEE31; MIM 617132)	nd	Chen et al. (2025)
RNAi	*DNM1*	Developmental and epileptic encephalopathy 44 (DEE44; MIM 616346)	nd	Aimiuwu et al. (2020); Jones et al. (2024)
ExSpeU1s	*CDKL5*	Developmental and epileptic encephalopathy 2 (DEE2; MIM 300203)	nd	Balestra et al. (2019)
saRNAs	*FOXG1*	Congenital variant of Rett syndrome (RTT variant; MIM 613454)	nd	Fimiani et al. (2016)

ASO, antisense oligonucleotides; AntagoNAT, antisense oligonucleotides that inhibit natural antisense transcripts; SINEUP, Synthetic Noncoding RNAs, that enhance translation by binding to the Untranslated (UP) region of target mRNA; RNAi, small RNA, interference; ExSpeU1s, Exon-Specific engineered U1 small nuclear RNAs; saRNAs, small activating RNA.

*CDKL5,* Cyclin-dependent kinase-like 5; *CHD8,* Chromodomain helicase DNA-binding protein 8; *DNM1,* Dynamin 1*; FMR1*, Fragile X messenger ribonucleoprotein1; *KCNT1,* potassium channel, subunit T member 1; *FOXG1*, Forkhead Box G1; *MECP2*, methyl-CpG-binding protein 2; *SCN1A,2A,8A*, Sodium Voltage-gated channel alpha subunit 1A,2A,8A; *UBE3A*, ubiquitin-protein ligase E3A; *UBA5*, Ubiquitin-like modifier-activating enzyme 5; nd, not determined.

## Antisense oligonucleotides (ASOs)-mediated targeting of FMR1, MECP2, SCN1A, SCN2A, SCN8A and KCNT1 transcripts

ASOs are short, single-stranded nucleic acid sequences typically ranging from 12 to 30 nucleotides in length. They bind to target cellular RNAs through sequence-specific complementary base pairing, enabling precise modulation of gene expression. They influence various RNA processes, including pre-mRNA splicing, mRNA stability, transcriptional regulation, and RNA-protein interactions (Crooke et al., 2021). A key advantage of ASOs lies in their high specificity for target sequences, which minimizes off-target effects and enhances safety profiles. This property allows ASOs to be designed for specific gene mutations, offering a precise therapeutic approach regulating disease-associated gene expression.

In Fragile X syndrome (FXS; MIM 300624), a leading cause of ID, ASO-mediated targeting of intron retention in *FMR1* transcripts effectively corrects abnormal alternative splicing and rescues FMRP expression in patient-derived cells (Shah et al., 2023). In induced pluripotent stem cell (iPSC)-derived cortical excitatory neurons isolated from patients with *MECP2* duplication syndrome (MDS; MIM 300260) and in humanized MDS mice, *MECP2*-ASO restores expression of MECP2-regulated genes in both disease systems. It also mitigates aberrant neuronal morphology in MDS neurons (Bajikar et al., 2024) and behavioral deficits in MDS mice (Shao et al., 2021). In Dravet syndrome (DS; MIM607208), a severe form of DEE which is primarily caused by *SCN1A* haploinsufficiency, treatment in a DS mouse model with the antisense oligonucleotide STK-001 (also known as ASO-22) increases the production of functional *Scn1a* mRNA and Nav1.1 protein. Mechanistically, STK-001 prevents the inclusion of the nonsense-mediated decay (NMD) exon 20N in the SCN1A gene, resulting in increased SCN1A transcript and protein levels (Han et al., 2020). Exon 20N acts as a “poison” exon, which, if included, triggers NMD and subsequent mRNA degradation. By binding to SCN1A pre-mRNA, STK-001 promotes skipping of exon 20N, allowing the production of full-length stable SCN1A mRNA. Through this mechanism, STK-001 corrects haploinsufficiency restoring functional Nav1.1 channel levels in a mouse model of Dravet syndrome with reduced electrographic seizures and sudden unexpected death in epilepsy (SUDEP) (Han et al., 2020). A clinical trial is currently underway in patients with Dravet syndrome (ClinicalTrials.gov ID: NCT04740476) to assess the effects of repeated STK-001 doses on seizure frequency and quality of life, as well as to evaluate long-term safety and tolerability. Recently, Yuan et al. (2024) also demonstrate the therapeutic potential of ASO-84 that is a surrogate of ASO-22 targeting a different *Scn1a* region with improved stability and binding affinity. The authors proved that a single injection of ASO-84 in DS mice brain leads to a robust upregulation of SCN1A protein, seizure reduction, and improved mice survival (Yuan et al., 2024).

Conversely, in DEE conditions associated with GoF mutations or toxic overexpression, ASOs are used to reduce the levels of pathogenic transcripts. As an example, in a mouse model of early-onset *SCN2A*-DEE (also named DEE11; MIM613721) driven by GoF mutation, administration of ASO targeting *Scn2a* mRNA led to decreased gene expression and improvement in seizure symptoms (Li et al., 2021). Importantly, therapeutic benefit was also observed in a preterm infant with early-onset *SCN2A*-DEE (Wagner et al., 2025), providing initial evidence in support of the ongoing clinical trial (ClinicalTrials.gov ID: NCT07019922). As well, in a mouse model of *SCN8A*-DEE (also named DEE13; MIM 614558) caused by a GoF mutation, long-term *Scn8a* downregulation by repeated treatments with *Scn8a* ASO provides long-term survival and reduced seizure frequency (Hill et al., 2024). Additionally, in a mouse model of *KCNT1*-DEE (also named DEE14; MIM614959), carrying a pathogenic *Kcnt1* variant, after a single intracerebroventricular bolus injection of *Kcnt1* ASO, at postnatal day 40, the mutant mice show a marked knockdown of *Kcnt1* mRNA with seizure frequency reduction, prolonged survival, and improved behavior (Burbano et al., 2022).

## Anti-natural antisense transcript (antagoNAT) inhibiting UBE3A-NAT transcript

AntagoNATs are a subclass of ASOs specifically engineered to inhibit natural antisense transcripts (NATs)—endogenous non-coding RNAs that are transcribed from the opposite DNA strand of protein-coding genes. These NATs often function as negative regulators of gene expression through diverse mechanisms, including transcriptional interference, RNA duplex formation, and chromatin modification. By selectively silencing these repressive NATs, antagoNATs can derepress gene expression and restore physiological protein levels, offering a targeted approach to modulate dosage-sensitive genes implicated in NDDs (Modarresi et al., 2012). This therapeutic strategy has shown promise in the context of Angelman syndrome (AS; MIM 105830), a severe NDD characterized by ID, epilepsy, ataxia, and lack of speech. AS is most caused by loss of function (LoF) of the maternally inherited *UBE3A* allele in neurons, where the paternal allele is epigenetically silenced by the long non-coding *UBE3A* antisense transcript (*UBE3A*-AS). Dindot et al. (2023) developed antagoNATs to repress *UBE3A*-AS transcription proving the reactivation of the paternal allele in iPSC-derived neurons from AS patients and in brain regions of cynomolgus monkey, a non-human primate model. In maternal *Ube3a* knockout mice, Lee et al. (2023) showed that a single antagoNAT dose restores paternal *Ube3a* allele and counteracts two prominent clinical AS features such as abnormal EEG rhythms and sleep disturbances. The importance of early administration of antagoNAT-based therapy is underscored by the authors as a key factor for therapeutic efficacy. Building on this, Clarke et al. (2024) showed that a single intracranial (IC) injection of antagoNATs delivered *in utero*, induces a reactivation of the paternal *Ube3a* allele in a AS mouse model lacking the maternal *Ube3a* gene. Treated AS mice exhibit increased UBE3A protein levels in both cortical and hippocampal neurons, along with improvements in motor function, cognitive performance, and seizure phenotype. This silencing strategy laid the foundation for clinical trials targeting paternal *UBE3A* reactivation (ClinicalTrials.gov ID: NCT04259281, NCT04428281 and NCT05127226).

## Short Interspersed Nuclear Element UP-regulating RNAs (SINEUPs) enhancing translation of CHD8 and UBA5 transcripts

SINEUPs represent a novel class of natural and synthetic antisense long non-coding RNAs (lncRNAs) that selectively enhance the translation of specific mRNAs without altering their transcript levels (Zucchelli et al., 2015). This unique mechanism relies on a modular, dual-domain structure with a target-specific antisense region that binds to the 5′ untranslated region (UTR) of the mRNA, and an effector domain derived from an inverted SINEB2 element, which facilitates the recruitment of ribosomes and promotes translation. By increasing ribosome occupancy and enhancing the association of target mRNAs with polysomes, SINEUPs offer a highly selective method to boost protein synthesis. This precision makes SINEUPs particularly attractive for therapeutic applications in genetic disorders with haploinsufficiency, where restoring normal protein level from a single functional allele can mitigate disease phenotype. A recent study by Di Leva et al. (2025) demonstrated the therapeutic potential of a synthetic SINEUP targeting *CHD8*, a risk gene for ASD and ID with macrocephaly (MIM 615032). In human iPSC–derived neural progenitor cells (hiNPCs) with *CHD8* knockdown and in fibroblasts derived from patients carrying *CHD8* nonsense or frameshift mutations, electroporation of SINEUP-*CHD8* led to increased levels of CHD8 protein. This upregulation partially rescues key molecular deficits, including transcriptional dysregulation and loss of H3K36me3 histone marks. In cortical organoids (COs) derived from patients with *UBA5*- DEE (also named DEE31; MIM 617132), which is caused by pathogenic variants resulting in UBA5 haploinsufficiency, a recent study provides evidence for the therapeutic potential of SINEUP in correcting *UBA5*-associated dysfunctions (Chen et al., 2025). *UBA5* encodes an E1-like activating enzyme essential for initiating the UFMylation pathway, a ubiquitin-like post-translational modification critical for maintaining endoplasmic reticulum (ER) proteostasis and neuronal homeostasis. Mutations in *UBA5* impair this pathway, leading to ER stress, disrupted proteostasis, and defective neuronal excitability—hallmark features of DEE31. To counteract the *UBA5* haploinsufficiency, the authors show that lentiviral-mediated delivery of SINEUP-*UBA5* into patient-derived DEE-COs significantly increases UBA5 protein expression along with reduction of ER stress response, partial recovery of neuronal firing and synaptic activity.

## Allele-specific RNA interference (RNAi) suppressing mutant DNM1 transcript

In the context of NDDs caused by GoF or dominant-negative mutations, allele-specific RNAi has emerged as a highly promising therapeutic strategy. This approach selectively suppresses the expression of the mutant allele preserving the wild-type allele, thereby preventing the production of dysfunctional or toxic proteins. Indeed, small interfering RNAs (siRNAs) or microRNAs (miRNAs) -designed to bind sequence specifically to mutant transcripts- can induce their degradation *via* the RNA-induced silencing complex and therefore halting translation. A powerful example is provided by *DNM1*-DEE (also named DEE44; MIM 616346), a severe early-onset DEE caused by dominant-negative mutations in *DNM1*, a gene encoding dynamin-1, a GTPase essential for synaptic vesicle recycling. In a mouse model of *DNM1*-DEE, Aimiuwu et al. (2020) demonstrated that a single neonatal injection of a miRNA targeting the mutant *Dnm1* transcript resulted in robust allele-specific knockdown. This intervention led to a significant reduction in seizure frequency and improvement in behavioral phenotype, underscoring the therapeutic potential of early postnatal RNAi delivery. Successively, Jones et al. (2024) employed a refined dual strategy in a conditional knock-in mouse model harboring the *Dnm1* G359A variant, which mimics a recurrent pathogenic mutation identified in DEE patients. The authors report on the functional outcome of the simultaneous delivery of an RNAi construct - to specifically silence the mutant *Dnm1* G359A allele- and of an exogenous wild-type *Dnm1* transgene -to restore total DNM1 protein levels. The combination of the allele-selective suppression coupled with the gene replacement effectively rebalances *DNM1* expression, preventing dominant-negative interference. As a result, treated *DNM1*-DEE mice exhibited substantial improvements in seizure burden, motor coordination, and exploratory behavior (Jones et al., 2024).

## Exon-specific engineering U1 small nuclear RNAs (ExSpeU1s) correcting CDKL5 splicing defects

Approximately half of the pathogenic variants implicated in NDDs are predicted to disrupt pre-mRNA splicing. A promising RNA-based strategy is focused on the correction of splicing defects by using the engineering of U1 small nuclear RNA (U1 snRNA), a core component of the spliceosome that recognizes and binds the 5′ splice site (5′ ss) during intron removal. By using this strategy, it is possible to rescue aberrant splicing events caused by mutations that weaken or eliminate canonical splice signals, thereby restoring correct mRNA processing and potentially mitigating the molecular pathology associated with such variants (Rogalska et al., 2016). Two main classes of engineered U1 snRNAs have been developed to address different types of splice-site mutations. The first involves modified U1 snRNAs, which are reprogrammed to enhance complementarity to mutated or suboptimal 5′ splice sites. These modified molecules help re-establish stable U1 snRNA–pre-mRNA interactions, enabling proper spliceosome assembly and accurate exon recognition (Gonçalves et al., 2023). The second, more innovative class consists of exon-specific U1 snRNAs (ExSpeU1s). Unlike canonical U1 snRNAs, ExSpeU1s do not bind directly to the 5′ splice site but hybridize to intronic regions downstream of the exon that is at high risk of being skipped. This strategic binding recruits splicing regulatory factors to the exon, promoting its inclusion into the mature mRNA transcript—even in the presence of a defective or absent 5′ splice site. By avoiding the dependency on canonical splice signals, ExSpeU1s expand the therapeutic scope of U1-based splicing correction, offering potential treatment avenues for a wide range of splice-disrupting mutations (Gonçalves et al., 2023). Given the ability to customize U1 snRNAs, several studies are actively exploring U1 snRNA-based therapies in models of NDDs. Proof-of-concept for this approach has been demonstrated in a study targeting *CDKL5*, a gene associated with early-onset DEE (also named DEE2; MIM 300203). By using engineered lentiviral ExSpeU1s, the authors successfully rescue the inclusion of exon 6 in *Cdkl5* null hippocampal neurons in which *Cdkl5* expression was disrupted by a splicing mutation affecting +5 nucleotides. As beneficial effects, the authors demonstrated the full restoration of CDKL5 protein synthesis and its proper subcellular distribution, along with the rescue of defective neuronal arborization, in comparison to neurons infected with the wild-type *Cdkl5* construct (Balestra et al., 2019). Recently, Sönmezler and colleagues (2024) explored the potential of modified U1snRNAs as a therapeutic strategy for splicing variants classified as *most likely pathogenic* in NDD genes, many of which are associated with syndromic ID, as *SCN2A* and *DDB1*. The authors employed a modified U1 snRNA approach to correct variant-induced splicing defects in HeLa cells, demonstrating partial rescue in four of the analyzed genes with correction efficiency varying across different gene targets (Sönmezler et al., 2024). Additional evidence for the therapeutic efficacy of ExSpeU1 is provided by its application in correcting splicing mutations in *OTC* gene detected in patients with ornithine transcarbamylase deficiency (OTCD; MIM 311250). This is an X-linked disease characterized by hyperammonemia and secondary neurodevelopmental impairments—such as encephalopathy and cognitive deficits—resulting from the toxic accumulation of ammonia in the brain (Balestra et al., 2020). Using AAV8-mediated delivery of ExSpeU1 in a murine model of OTCD carrying the c.386G>A splice-site mutation, Balestra et al. (2020) demonstrated a significant increase in correctly spliced *OTC* transcripts, highlighting the *in vivo* potential of targeted splicing correction and its future relevance in preventing neurological complications.

## Small activating RNA (saRNAs) correcting the defective FOXG1 haploinsufficiency

Many genes that play essential roles in brain development and function are dosage-sensitive, meaning that their proper expression requires both gene copies. Hemizygous deletions can affect genes that are critical for neuronal development, synaptic plasticity, and neuronal signaling pathways, leading to a variety of NDDs, including ID, DEE, and ASD. Ongoing research is focused on developing saRNAs to upregulate the expression of the remaining functional allele to compensate for the missing copy. They are a class of small, double-stranded RNA molecules that are designed to target promoter regions of specific genes that can positively and reversibly upregulate their target genes above endogenous level (Ghanbarian et al., 2021; Roth et al., 2023). The mode of action for saRNA is dependent on the complementarity at the 5′-region of the antisense or guide strand oligo to the intended target DNA near or within gene promoters.

The pioneering study by Diodato et al. (2013) explored the use of saRNAs to transactivate the *Emx2* transcription factor gene in embryonic cortico-cerebral precursor cells to offset defective neuronogenesis. Successively, Fimiani et al. (2016) underscore the potential of saRNA technology to stimulate the *Foxg1* transcription factor gene, a key regulator of cortico-cerebral development and function correcting haploinsufficient conditions. Located on chromosome 14q13, heterozygous truncating mutations in the *FOXG1* gene have been found in patients with the congenital variant of Rett syndrome (MIM 613454). This is a severe NDD with features of classic Rett syndrome (RTT; 312750) - including ID, motor development, typical stereotypic hand and mouth movements- but earlier onset in the first months of life (Hou et al., 2020). Fimiani and collaborators (2016) identified a set of artificial miRNA capable stimulating *Foxg1* transactivation, specifically in cortico-cerebral cells. Interestingly, one of these miRNAs (miR-αFoxg1.1694) administrated to mouse neonates by intraventricular injection of recombinant AAV vectors, replicates *in vivo* the stimulation of *Foxg1*-RNA activation (Fimiani et al., 2016). Although applications in the NDD field are currently limited, saRNAs represent a promising activating RNA-based technology for targeting disease gene mitigating pathogenic signaling pathways.

## Conclusion

mRNA therapeutics have emerged as a transformative modality for the treatment of diverse diseases and conditions. This review outlines current RNA-based therapeutic strategies under investigation for NDDs, highlighting proof-of-concept studies that demonstrate their potential to correct specific molecular defects and enable more precise, personalized treatments. Following the successful development of mRNA-based therapies in neurodegenerative and cardiovascular diseases and oncology, this field continues to expand significantly, as reflected by the increasing number of scientific publications and growing investment. Very importantly, the great potential of these approaches could truly transform healthcare by addressing previously untreatable conditions and improving patient outcomes. A prominent example is ASO therapy with Nusinersen for Spinal Muscular Atrophy (SMA; MIM 253300) caused by mutations in the SMN1 gene on chromosome 5 (Finkel et al., 2017). SMA is a rare autosomal recessive neuromuscular degenerative disease characterized by loss of spinal cord motor neurons leading to progressive muscle wasting (Finkel et al., 2017). Nusinersen is the first FDA-approved ASO drug for patients in all stages of life capable to improve clinically the motor function deficits occurring in SMA patients (Finkel et al., 2017). It works by modulating the splicing of the SMN1 paralog, SMN2, promoting inclusion of exon 7 and enhancing functional SMN protein expression. It is available as an injection administered directly to the central nervous system intrathecally by a trained healthcare provider (Finkel et al., 2017).

Another notable example is patisiran, an FDA-approved siRNA therapy for patients with hereditary transthyretin-mediated (hATTR; MIM 105210) amyloidosis, a progressive cardiomyopathy caused by heterozygous mutations in the TTR gene (Maurer et al., 2023). The siRNA Patisiran works by targeting and degrading mutant TTR mRNA, thereby reducing the production of misfolded TTR protein and alleviating disease symptoms in hATTR patients (Maurer et al., 2023). Unlike SMA or hATTR, where they are FDA-approved, RNA-based therapies in oncology are mostly experimental or in trials (Çakan et al., 2024). One promising RNA-based drug is the ASO AZD8701 to selectively reduce human forkhead box P3 (FOXP3) expression in regulatory T cells, reversing their immunosuppressive function. Recently, it has been reported the first-in-human phase I study designed to define the applicability of AZD8701 in patients with advanced solid tumors, alone or in combination with durvalumab, to inform decision-making regarding monotherapy and anti–PD-(L)1 agent combinations in later-phase clinical studies (Siu et al., 2025).

Although the number of studies specifically focused on RNA therapeutics for NDDs remains limited, the field is rapidly evolving, with preclinical and early-stage clinical trials currently underway that aim to tailor RNA therapies to individual gene mutations. This is primarily attributable to the numerous advantages associated with this approach. For instance, unlike gene editing, RNA therapies offer reversible effects, which can be advantageous in fine-tuning treatment or minimizing long-term risks. Unlike conventional gene replacement therapies, RNA-based methods do not introduce exogenous gene copies reducing the risk of overexpression and associated toxicity. In addition, recognizing the crucial need to block pathogenic mechanisms as early as possible, recent progress in the early delivery of RNA therapeutics is drawing attention. Indeed, NDDs with early onset—such as ID, ASD, and DEEs—are increasingly being targeted by early-phase clinical trials, several of which are already underway and yielding promising preliminary results. Remarkably, they generally provide scalable systems making them suitable for rescuing dosage-sensitive protein defects.

However, advances in delivery systems, molecular targeting, and safety profiling are essential to translate these innovative approaches into effective clinical treatments for precision molecular intervention. One notable limitation of RNA-based therapies is the potential for off-target binding, which can inadvertently modulate the expression of unplanned genes leading to adverse biological effects. Because most RNA therapies require repeated administration to sustain therapeutic efficacy, there is a risk of cumulative toxicity or off-target accumulation, particularly in organs such as liver and kidney. Furthermore, although intrathecal administration and viral vector–based delivery systems facilitate targeting of the central nervous system, their invasive nature and associated risks highlight the pressing need for safer and more efficient delivery strategies—particularly considering the restrictive characteristics of the blood–brain barrier. Lastly, it is essential to consider that genetic background and disease stage may contribute to interindividual variability, which can significantly affect therapeutic response and hinder efforts toward standardizing treatment protocols. Nevertheless, although significant challenges remain in advancing RNA-based therapies for NDD conditions, recent studies highlighted in this review illustrate the potential of RNA-based approaches to modulate NDD-driving pathways. Further studies in this direction are essential to enable the development of increasingly precise and effective therapies for incurable pediatric disorders.
